# *N*,*N′*,*N′′*-Triethylhexahydro-1,3,5-triazin: MAK-Begründung, Nachtrag

**DOI:** 10.34865/mb777927kskd10_2ad

**Published:** 2025-06-30

**Authors:** Andrea Hartwig, MAK Commission

**Affiliations:** 1 Institut für Angewandte Biowissenschaften. Abteilung Lebensmittelchemie und Toxikologie. Karlsruher Institut für Technologie (KIT) Adenauerring 20a, Geb. 50.41 76131 Karlsruhe Deutschland; 2 Ständige Senatskommission zur Prüfung gesundheitsschädlicher Arbeitsstoffe. Deutsche Forschungsgemeinschaft, Kennedyallee 40, 53175 Bonn, Deutschland. Weitere Informationen: Ständige Senatskommission zur Prüfung gesundheitsschädlicher Arbeitsstoffe | DFG

**Keywords:** N,N′,N′′-Triethylhexahydro-1,3,5-triazin, Nase, Reizwirkung, Kanzerogenität, Formaldehydabspalter, Keimzellmutagenität, 1,3,5-triethylhexahydro-1,3,5-triazine, nose, irritation, carcinogenicity, formaldehyde releaser, germ cell mutagenicity

## Abstract

The German Senate Commission for the Investigation of Health Hazards of Chemical Compounds in the Work Area (MAK Commission) has re-evaluated 1,3,5-triethylhexahydro-1,3,5-triazine [7779-27-3] with regard to its carcinogenicity and germ cell mutagenicity classification, its ability to be absorbed through the skin, its sensitization potential and whether an occupational exposure limit value (maximum concentration at the workplace, MAK value) can be derived. Relevant studies were identified from a literature search. 1,3,5-Triethylhexahydro-1,3,5-triazine is corrosive to the skin and eyes. The substance is a formaldehyde releaser and is expected to undergo rapid hydrolysis in aqueous solution. For this reason, the local irritation is attributed to the hydrolysis products formaldehyde and ethylamine. Carcinogenicity, toxicity and genotoxicity of 1,3,5-﻿triethylhexahydro-1,3,5-triazine in the upper respiratory tract or nose, the likely target organs, have not been investigated. The substance has mutagenic and clastogenic potential in vitro, presumably due to the release of formaldehyde. Formaldehyde was classified in Carcinogen Category 4 because it induces tumours in nasal tissues at concentrations that exceed their detoxification capacity. As a formaldehyde releaser, 1,3,5-triethylhexahydro-1,3,5-triazine could likewise be classified in Carcinogen Category 4. However, because it is not possible to derive a MAK value for 1,3,5-triethylhexahydro-1,3,5-triazine, the substance has been assigned to Carcinogen Category 2 and given the footnote “Prerequisite for Category 4 in principle fulfilled, but insufficient data available for the establishment of a MAK or BAT value”. As there are no data for the systemic bioavailability of 1,3,5-triethylhexahydro-1,3,5-triazine and the formaldehyde that is released by hydrolysis in tissues, there is no experimental evidence that the formaldehyde reaches the germ cells. Therefore, 1,3,5-triethylhexahydro-1,3,5-triazine has been classified in Category 3 B for germ cell mutagens. There is still no evidence for a sensitizing potential of 1,3,5-triethylhexahydro-1,3,5-triazine. Skin contact is not expected to contribute significantly to systemic toxicity.

**Table:** 

**MAK-Wert**	**–**
**Spitzenbegrenzung**	**–**
	
**Hautresorption**	**–**
**Sensibilisierende Wirkung**	**–**
**Krebserzeugende Wirkung (2024)**	**Kategorie 2^a)^**
**Fruchtschädigende Wirkung**	**–**
**Keimzellmutagene Wirkung (2024)**	**Kategorie 3 B**
	
**EKA**	–
	
Synonyma	Hexahydro-1,3,5-triethyl-1,3,5-triazin 1,3,5-Triethylhexahydro-1,3,5-triazin 1,3,5-Triethylhexahydro-*s*-triazin 1,3,5-Triethyl-1,3,5-triazacyclohexan Triethyltrimethylentriamin
Chemische Bezeichnung (IUPAC-Name)	1,3,5-Triethyl-1,3,5-triazinan
CAS-Nr.	7779-27-3
Formel	Strukturformel von N,N′,N′′-Triethylhexahydro-1,3,5-triazin. 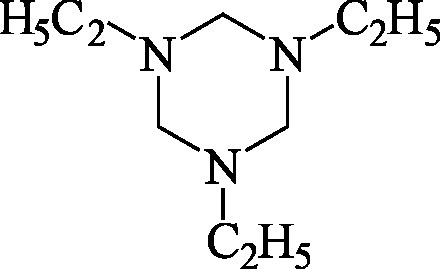
	C_9_H_21_N_3_
Molmasse	171,29 g/mol
log K_OW_	0,74 (ber., Mittelwert; US EPA 2024)
Siedepunkt bei 1013 hPa	207–208 °C (LookChem 2023), 205–210 °C (Greim 2003; US EPA 1997)
Dampfdruck	21 hPa bei Raumtemperatur (Greim 2003), vermutlich Dampfdruck von Wasser aus einer wässrigen Lösung des Produkts 0,319 hPa bei 25°C (LookChem 2023), 0,114 hPa bei 25°C (ber.; US EPA 2022)
pH-Wert	ca. 11 als 5%ige wässrige Lösung (US EPA 1997)
**1 ml/m^3^ (ppm) ≙ 7,107 mg/m^3^**	**1 mg/m^3^ ≙ 0,141 ml/m^3^ (ppm)**
Hydrolysestabilität	Zerfall in Formaldehyd und Ethylamin bei pH-Werten kleiner als 6,3 (k. w. A.; Greim 2003), stabil im basischen Bereich (US EPA 1997)
Einsatzverbote	Der Stoff ist im Rahmen von REACH lediglich vorregistriert (ECHA 2023). Es gibt weder ein REACH-Dossier, noch eine Biozid-Anmeldung, sodass der Stoff derzeit weder importiert, produziert noch verwendet werden dürfte.

^a)^ Voraussetzung für Kategorie 4 prinzipiell erfüllt, aber Daten für MAK- oder BAT-Wert-Ableitung nicht ausreichend

Hinweis: Formaldehydabspalter. Kann gleichzeitig als Dampf und Aerosol vorliegen.

Es liegt eine Begründung (Greim 2003) vor. Es erfolgt eine Neubewertung der Daten zur Formaldehydabspaltung nach aktuellem Vorgehen der Kommission (DFG 2024). Unter diesem Aspekt werden in diesem Nachtrag die bewertungsrelevanten Endpunkte re-evaluiert.

## Allgemeiner Wirkungscharakter

*N*,*N′*,*N′′*-Triethylhexahydro-1,3,5-triazin ist ein Formaldehydabspalter. Der vermutlich kritische Effekt von *N*,*N′*,*N′﻿′﻿*-﻿Triethylhexahydro-1,3,5-triazin ist die kanzerogene und reizende Wirkung von freigesetztem Formaldehyd am Atemtrakt. Der Stoff wirkt ätzend auf Haut, Augen und Schleimhäute. Bei epikutaner Applikation ist noch die niedrigste getestete Dosis von 10 mg/kg KG und Tag bei Ratten hautreizend. Systemische Wirkungen sind nach 13-wöchiger oraler und epikutaner Applikation an Ratten bis 100 mg/kg KG und Tag nicht beschrieben. *N*,*N′*,*N′′*-Triethylhexahydro-1,3,5-tria﻿zin ist in vitro genotoxisch im Salmonella-Mutagenitätstest, im UDS-Test an Rattenhepatozyten sowie im HPRT-Genmutationstest an CHO (Chinese Hamster Ovary)-Zellen, nicht jedoch in vivo im Maus-Mikronukleustest an Knochenmarkszellen.

## Wirkungsmechanismus

*N*,*N′*,*N′′*-Triethylhexahydro-1,3,5-triazin ist ein Formaldehydabspalter. Formaldehyd zeigt deutliche Reizwirkungen in der Nase und kann bei chronischer Exposition Tumoren im Epithel der Nasenschleimhaut erzeugen. Die Entgiftungskapazitäten des Nasengewebes für Formaldehyd werden bei einem MAK-Wert von 0,3 ml/m^3^ (0,37 mg/m^3^) nicht überschritten (Greim 2000; Hartwig 2010). Sein Dampfdruck beträgt 5185 hPa (OECD 2002).

Es liegen verschiedene Daten zum Dampfdruck von *N*,*N′*,*N′′*-Triethylhexahydro-1,3,5-triazin vor: 21 hPa (Greim 2003, vermutlich Dampfdruck von Wasser), 0,319 hPa (LookChem 2023) und 0,114 hPa bei 25°C (ber., US EPA 2022). Aufgrund des hohen Siedepunktes des Stoffs im Bereich von 205–210 °C (Greim 2003; LookChem 2023; US EPA 1997) ist ein niedriger Dampfdruck als eher wahrscheinlich anzusehen. Dieser und der Siedepunkt sprechen dafür, dass *N*,*N﻿′*,*N﻿′﻿′*-﻿Triethylhexahydro-1,3,5-triazin als Dampf/Aerosol-Gemisch vorliegen kann (DFG 2024). Für Aerosole von Formaldehydabspaltern ist durch die Impaktierung im Atemtrakt mit einer stärkeren Wirkung als der durch dampfförmigen Formaldehyd verursachten Wirkung zu rechnen. Dies wurde mit einem anderen Formaldehydabspalter, zu dem eine Inhalationsstudie vorliegt, gezeigt (siehe Begründung *N*,*N′*,*N′′*-Tris(β-hydroxyethyl)hexahydro-1,3,5-triazin; Hartwig und MAK Com﻿mis﻿sion 2023).

## Toxikokinetik und Metabolismus

Über die Stabilität und Zerfallskinetik liegen keine verlässlichen Angaben vor, außer dass *N*,*N′*,*N′′*-Triethylhexahy﻿dro-1,3,5-triazin bei pH-Werten unter 6,3 in jeweils drei Moleküle Formaldehyd und Ethylamin zerfällt (Greim 2003).

Zur perkutanen Resorption liegen keine experimentellen Untersuchungen vor. Modellrechnungen nach IH Skin﻿Perm v2.04 (Tibaldi et al. 2014) und Fiserova-Bergerova et al. (1990) liefern für eine Stoffkonzentration von 1 % (angenomme﻿ne nicht mehr reizende Konzentration für einen ätzenden Stoff) in wässriger Lösung unter Standardbedingungen (60 Minuten Expositionsdauer, 2000 cm^2^ exponierte Hautfläche) unter Verwendung eines log K_OW_ von 0,74 eine Resorption von 12,9 bzw. 75,6 mg. Nicht berücksichtigt wurde dabei eine zu vermutende rasche Hydrolyse des Stoffes im sauren Milieu der Hautoberfläche, die einer Resorption der unzersetzten Verbindung entgegenwirkt.

## Tierexperimentelle Befunde und In-vitro-Untersuchungen

Daten zur akuten und chronischen Toxizität, zur Wirkung auf Haut unnd Schleimhäute sowie zur Entwicklungstoxizität sind in Greim (2003) dargestellt.

### Allergene Wirkung

Neue Daten zur sensibilisierenden Wirkung liegen nicht vor.

### Genotoxizität

Neue Daten zur genotoxischen Wirkung liegen nicht vor.

#### In vitro

*N*,*N′*,*N′′*-Triethylhexahydro-1,3,5-triazin (Reinheit 98 %) zeigte sich in vitro an Bakterien mutagen und war genotoxisch im UDS-Test an Rattenhepatozyten sowie im HPRT-Genmutationstest an Säugetierzellen (Greim 2003).

#### In vivo

*N*,*N′*,*N′′*-Triethylhexahydro-1,3,5-triazin (Reinheit 98 %) wirkte nicht genotoxisch in vivo im Maus-Mikronukleustest an Knochenmarkszellen bei oraler Gabe. Im Hinblick auf die berichtete Instabilität von *N*,*N′*,*N′′*-Triethylhexahy﻿dro-1,3,5-triazin im sauren pH-Bereich konnte nicht ausgeschlossen werden, dass dieses bereits im Magen zerfiel und Formaldehyd dort abreagierte und das Knochenmark nicht erreichte (Greim 2003).

Auch Formaldehyd zeigte nur mit hohen intraperitonealen Dosierungen positive Effekte im Maus-Mikronukleustest, nicht aber nach oraler Gabe (Greim 2000), sodass aufgrund des negativen oralen In-vivo-Tests eine keimzellmutagene Wirkung von *N*,*N′*,*N′′*-Triethylhexahydro-1,3,5-triazin nach Inhalation nicht völlig ausgeschlossen werden kann.

### Kanzerogenität

Hierzu liegen keine Daten vor.

## Bewertung 

Kritische Effekte sind die kanzerogene und lokal reizende Wirkung des Hydrolyseprodukts Formaldehyd.

**Krebserzeugende Wirkung. **Es liegen keine Untersuchungen der krebserzeugenden Wirkung von *N*,*N′*,*N′′*-Triethylhexahydro-1,3,5-triazin vor. *N*,*N′*,*N′′*-Triethylhexahydro-1,3,5-triazin selbst weist in den vorliegenden Tests nur in vitro ein genotoxisches Potenzial auf, eine mögliche genotoxische Wirkung am wahrscheinlichen Zielgewebe Nase (wie bei Formaldehyd) ist jedoch nicht untersucht.

Kanzerogenitätsstudien zum Zerfallsprodukt Ethylamin gibt es nicht, das strukturverwandte Diethylamin ist bei Inhalation nicht kanzerogen (Hartwig und MAK Commission 2016).

Die lokale Kanzerogenität des Hydrolyseprodukts Formaldehyd hingegen ist ausführlich dokumentiert (Greim 2000; Hartwig 2010). Formaldehyd ruft Nasentumoren hervor, jedoch erst bei Konzentrationen, die die Entgiftungskapazitäten des Nasengewebes überschreiten. Daher ist Formaldehyd in Kanzerogenitäts-Kategorie 4 eingestuft.

Die Hydrolysegeschwindigkeit von *N*,*N′*,*N′′*-Triethylhexahydro-1,3,5-triazin ist unbekannt. Daher wird für die Bewertung am Arbeitsplatz bei inhalativer Exposition vom Worst-Case der sofortigen vollständigen Formaldehydfreisetzung ausgegangen. Deshalb könnte *N*,*N′*,*N′′*-Triethylhexahydro-1,3,5-triazin in Analogie zu Formaldehyd in Kanzerogenitäts-Kategorie 4 eingestuft werden.

Da jedoch kein MAK-Wert für *N*,*N′*,*N′′*-Triethylhexahydro-1,3,5-triazin abgeleitet werden kann, wird der Stoff der Kanzerogenitäts-Kategorie 2 zugeordnet und erhält die Fußnote „Voraussetzung für Kategorie 4 prinzipiell erfüllt, aber Daten für MAK- oder BAT-Wert-Ableitung nicht ausreichend“.

**MAK-Wert. **Es liegen keine Inhalationsstudien mit *N*,*N′*,*N′′*-Triethylhexahydro-1,3,5-triazin am Menschen oder am Tier vor, aus denen ein MAK-Wert abgeleitet werden kann.

*N*,*N′*,*N′′*-Triethylhexahydro-1,3,5-triazin hydrolysiert zu Formaldehyd und Ethylamin. Aufgrund der schnellen Hydrolyse bei leicht saurem pH-Wert und dem Fehlen genauer Daten wird für die Risikobewertung hinsichtlich einer Worst-Case-Abschätzung davon ausgegangen, dass *N*,*N′*,*N′′*-Triethylhexahydro-1,3,5-triazin beim Auftreffen im Atemtrakt sofort vollständig zu Formaldehyd und Ethylamin hydrolysiert und der Abbau des aus *N*,*N′*,*N′′*-Triethylhexahy﻿dro-1,3,5-triazin entstehenden Formaldehyds langsamer erfolgt als dessen Bildung.

Formaldehyd zeigt deutliche Reizwirkungen in der Nase und kann bei chronischer Exposition Tumoren im Epithel der Nasenschleimhaut erzeugen. Die Entgiftungskapazitäten des Nasengewebes für Formaldehyd werden bei einem MAK-Wert von 0,3 ml/m^3^ (0,37 mg/m^3^) nicht überschritten (Greim 2000; Hartwig 2010) und sein Dampfdruck beträgt 5185 hPa (OECD 2002).

Der MAK-Wert für Ethylamin beträgt 5 ml/m^3^ und der Dampfdruck 990 hPa bei 20 °C. Der kritische Effekt ist eine Reizung des Nasenepithels von Ratten mit einer NOAEC von 100 ml/m^3^ in einer 24-wöchigen Inhalationsstudie (Hartwig und MAK Commission 2019).

Es liegen verschiedene Daten zum Dampfdruck von *N*,*N′*,*N′﻿′*-﻿Triethylhexahydro-1,3,5-triazin vor: 21 hPa (Greim 2003), vermutlich der Dampfdruck von Wasser, 0,319 hPa (LookChem 2023) und 0,114 hPa bei 25 °C (ber., US EPA 2022). Aufgrund des hohen Siedepunktes des Stoffs im Bereich von 205–210 °C (Greim 2003; LookChem 2023; US EPA 1997) ist ein niedriger Dampfdruck als eher wahrscheinlich anzusehen. Dieser und der Siedepunkt sprechen dafür, dass *N*,*N﻿′*,*N﻿′﻿′*-﻿Triethylhexahydro-1,3,5-triazin als Dampf/Aerosol-Gemisch vorliegen kann (DFG 2024, S. 15). Dies ist auch bei einer Konzentration, die dem MAK-Wert von Formaldehyd entspricht, nicht auszuschließen.

Für Aerosole von Formaldehydabspaltern ist durch die Impaktierung im Atemtrakt mit einer stärkeren Wirkung, als der durch dampfförmigen Formaldehyd verursachten Wirkung, zu rechnen, wie mit einem anderen Formaldehydabspalter, zu dem eine Inhalationsstudie vorliegt, gezeigt wurde (siehe Begründung *N*,*N′*,*N′′*-Tris(β-hydroxyethyl)hexahydro-1,3,5-triazin; Hartwig und MAK Commission 2023).

Für *N*,*N′*,*N′′*-Triethylhexahydro-1,3,5-triazin kann somit kein MAK-Wert festgesetzt werden. Eine Spitzenbegrenzung entfällt.

Bei Anwendung in verdünnten wässrigen Lösungen sollte mit einer vollständigen Hydrolyse gerechnet und daher der MAK-Wert für Formaldehyd (Greim 2000; Hartwig 2010) eingehalten werden.

**Fruchtschädigende Wirkung. **Da kein MAK-Wert abgeleitet wird, entfällt die Zuordnung zu einer risikobasierten Schwangerschaftsgruppe. In einer pränatalen Entwicklungstoxizitätsstudie ähnlich der OECD-Prüfrichtlinie 414 wurde je 25 Sprague-Dawley-Ratten pro Gruppe 0, 10, 75 oder 150 mg *N*,*N′*,*N′′*-Triethylhexahydro-1,3,5-triazin/kg KG und Tag vom 6. bis zum 15. Gestationstag per Gavage verabreicht. Die Muttertiere zeigten bei der höchsten Dosis einen statistisch signifikanten Rückgang der Gewichtszunahme von 35 % während der Behandlungszeit und von 28 % für die gesamte Studiendauer unter Berücksichtigung des graviden Uterusgewichtes. Bei den Feten zeigten sich dosisabhängige Zunahmen von Variationen an den Harnleitern (bilateral erweitert und geknäuelt) und den Nieren (bilateral erweiterte Nierenbecken), die aber nur bei 150 mg/kg KG und Tag statistisch signifikant waren. Das Körpergewicht der Feten wurde durch die Behandlung nicht beeinträchtigt. Der NOAEL für Entwicklungs- und Maternaltoxizität lag bei 75 mg/﻿kg KG und Tag (Greim 2003). Auch wenn die Variationen an Harnleitern und Nieren nur bei einer maternaltoxischen Dosis auftreten, deutet das Ausbleiben eines Effektes auf das fetale Körpergewicht darauf hin, dass es sich nicht um eine behandlungsbedingte Entwicklungsverzögerung als Sekundäreffekt durch die Maternaltoxizität handelt. Die Studie liegt in gekürzter Form vor (26 von 275 Seiten), sodass eine genaue Analyse des Einflusses der Maternaltoxizität auf die Entwicklungstoxizität nicht möglich ist. Ein Verdacht auf eine entwicklungstoxische Wirkung kann daher nicht begründet werden.

**Keimzellmutagene Wirkung. ***N*,*N′*,*N′′*-Triethylhexahydro-1,3,5-triazin ist in vitro genotoxisch im Salmonella-Mutagenitätstest, im UDS-Test sowie im HPRT-Genmutationstest an Säugetierzellen, nicht jedoch in vivo im Maus-Mikronukleustest an Knochenmarkszellen. Diese genotoxischen Effekte stimmen mit den für Formaldehyd beobachteten überein. Untersuchungen an Keimzellen liegen mit *N*,*N′*,*N′′*-Triethylhexahydro-1,3,5-triazin nicht vor.

Formaldehyd ist in Kategorie 5 für Keimzellmutagene eingestuft. Dies bedeutet, dass bei Einhaltung des MAK-Wertes von 0,3 ml/m^3^ nur ein sehr geringer Beitrag zum genetischen Risiko für den Menschen zu erwarten ist (Greim 2000).

*N*,*N′*,*N′′*-Triethylhexahydro-1,3,5-triazin könnte zwar in Analogie zu Formaldehyd in die Kategorie 5 für Keimzellmutagene eingestuft werden, jedoch kann für *N*,*N′*,*N′′*-Triethylhexahydro-1,3,5-triazin kein MAK-Wert aufgestellt werden.

Da Daten zur systemischen Bioverfügbarkeit von *N*,*N′*,*N′′*-Triethylhexahydro-1,3,5-triazin und dem durch Hydrolyse freigesetzten Formaldehyd fehlen, liegt kein experimenteller Beleg vor, dass der freigesetzte Formaldehyd in aktiver Form die Keimzellen erreicht. Daher wird *N*,*N′*,*N′′*-Triethylhexahydro-1,3,5-triazin in Kategorie 3 B für Keimzellmutagene eingestuft.

**Hautresorption. **Zur dermalen Aufnahme von *N*,*N′*,*N′′*-Triethylhexahydro-1,3,5-triazin liegen keine experimentellen Untersuchungen vor. Abschätzungen auf der Basis von Modellrechnungen lassen unter Standardbedingungen eine Resorption von maximal 75,6 mg (0,441 mmol) aus einer 1%igen Lösung erwarten. Unter der Annahme einer raschen Hydrolyse des *N*,*N′*,*N′′*-Triethylhexahydro-1,3,5-triazins mit Bildung von Formaldehyd lässt sich die Zunahme des Formaldehydspiegels im Blut nach dermaler Applikation abschätzen: aus den zuvor berechneten insgesamt 0,37 mmol werden 3 × 0,441 mmol Formaldehyd = 39,8 mg Formaldehyd innerhalb einer Stunde freigesetzt, pro Minute demnach etwa 663 µg bzw. 828 µg/1,25 Minuten (mittlere Halbwertszeit des Formaldehyds im Blut; Kaden et al. 2010). Die transdermale Aufnahme von *N*,*N′*,*N′′*-Triethylhexahydro-1,3,5-triazin in den letzten sechs Halbwertszeitintervallen führt demnach zu einer im Blut zirkulierenden Formaldehydmenge von (828 + 414 + 207 + 104 + 52 + 26) µg = 1,63 mg.

Der physiologisch bedingte Formaldehydspiegel im Blut des Menschen beträgt etwa 2–3 mg/l (10–15 mg in 5 l Blut) (Heck et al. 1985). Der zusätzliche Eintrag durch transdermal aufgenommenes *N*,*N′*,*N′′*-Triethylhexahydro-1,3,5-triazin erhöht demnach die Gleichgewichtskonzentration von Formaldehyd im Blut unbedeutend, sodass der Stoff weiterhin nicht mit „H“ markiert wird.

**Sensibilisierende Wirkung. **Zur sensibilisierenden Wirkung beim Menschen liegen nach wie vor keine Erfahrungen vor. Ferner stehen keine neuen tierexperimentellen Untersuchungen oder Daten aus tierversuchsfreien Alternativverfahren zur Verfügung. Es erfolgt daher weiterhin keine Markierung mit „Sh“ oder „Sa“.
